# Encapsulation of Activated Carbon into a Hollow-Type Spherical Bacterial Cellulose Gel and Its Indole-Adsorption Ability Aimed at Kidney Failure Treatment

**DOI:** 10.3390/pharmaceutics12111076

**Published:** 2020-11-11

**Authors:** Toru Hoshi, Masahito Endo, Aya Hirai, Masashige Suzuki, Takao Aoyagi

**Affiliations:** 1 Department of Materials and Applied Chemistry, College of Science and Technology Nihon University, 1-8-14, Kanda-Surugadai, Chiyoda-ku, Tokyo 101-8308, Japan; aoyagi.takao@nihon-u.ac.jp; 2 Department of Materials and Applied Chemistry, Graduate School of Science and Technology, Nihon University, 1-8-14, Kanda-Surugadai, Chiyoda-ku, Tokyo 101-8308, Japan; csma18005@g.nihon-u.ac.jp (M.E.); csay15163@g.nihon-u.ac.jp (A.H.); csma19025@g.nihon-u.ac.jp (M.S.)

**Keywords:** bacterial cellulose, encapsulation, hollow sphere, activated carbon, indole adsorption

## Abstract

For reducing side effects and improvement of swallowing, we studied the encapsulation of activated carbon formulations with a hollow-type spherical bacterial cellulose (HSBC) gel using two kinds of encapsulating methods: Methods A and B. In Method A, the BC gelatinous membrane was biosynthesized using *Komagataeibacter xylinus* (*K. xylinus*) at the interface between the silicone oil and cell suspension containing activated carbon. In Method B, the bacterial cellulose (BC) gelatinous membrane was formed at the interface between the cell suspension attached to the alginate gel containing activated carbon and the silicone oil. After the BC gelatinous membrane was biosynthesized by *K. xylnus*, alginate gel was removed by soaking in a phosphate buffer. The activated carbon encapsulated these methods could neither pass through the BC gelatinous membrane of the HSBC gel nor leak from the interior cavity of the HSBC gel. The adsorption ability was evaluated using indole, which is a precursor of the uremic causative agent. From curve-fitting, the adsorption process followed the pseudo-first-order and intra-particle diffusion models, and the diffusion of the indole molecules at the surface of the encapsulated activated carbon within the HSBC gel was dominant at the initial stage of adsorption. It was observed that the adsorption of the encapsulated activated carbon by the intraparticle diffusion process became dominant with longer adsorption times.

## 1. Introduction

The kidney acts as a filter for all of the fluids in our body. Kidney failure is characterized by an accumulation of human metabolic products (uremic toxins) in the blood as it is then impossible to eliminate them [1]. The artificial removal of these toxins is generally achieved by blood purification or dialysis. Chronic kidney disease (CKD) is a serious healthcare problem, which can lead to end-stage renal disease (ESRD) requiring dialysis or kidney transplantation [2].

AST-120 (KREMEZIN^®^) is an orally-administered spherical carbon adsorbent consisting of porous carbon particles that are 0.2–0.4 mm in diameter and insoluble in water and that can adsorb various small molecules, including uremic toxins. AST-120 treats uremic symptoms and prolongs the time to initiation of dialysis in patients with progressive CKD [3]. However, carbon adsorbents such as AST-120 and activated carbon powder are difficult to handle for patients [4]. These oral carbon absorbents have disadvantages, such as a gritty feeling, stickiness in the mouth and the throat in swallowing, large tablet size, and large dosage. To reduce the dose of carbon absorbent, it is desirable to use a carbon absorbent with a smaller particle size and larger surface area. However, a carbon absorbent with such characteristics has a great ability to adhere onto the intestinal wall, and carbon absorbents adsorbed on the intestinal wall have been reported to cause serious side effects such as intestinal obstruction and perforation [5,6,7]. There is a limit to the possible reduction of the particle size of carbon adsorbents; therefore, it is necessary to develop a carbon adsorbent drug that is easy to swallow and effective at a small dosage.

Nakano et al. reported that when agar gel beads encapsulated activated carbon with a particle size of 61–295 µm as an oral adsorbent, the capsulated activated-carbon beads were retained within the spherical agarose gel beads [4]. Encapsulation with agarose gel did not impair the adsorption capacity of the activated carbon, and these agar-encapsulated activated-carbon beads demonstrated the same adsorption capacity and adsorption behavior as AST-120. The encapsulation of activated-carbon particles is expected to prevent side effects by preventing direct contact between activated carbon and the intestinal wall.

Encapsulation technology has been studied in various application fields, such as pharmaceuticals, food sciences, paints, cosmetics, and adhesives. Specifically, the encapsulation of islet cells with alginate has been demonstrated to be effective in treatment for type I diabetes [8,9], but there are problems with biocompatibility. Improvement of the biocompatibility of alginate microcapsules requires a reduction in impurities [10] and an increase in the composition of guluronic acid [11]. Therefore, alternative microencapsulated materials to alginate have been explored, including polyethylene glycol, poly(methyl methacrylate), agarose, chitosan, collagen, and gelatin [12,13,14].

Many types of natural and synthetic polymers have been investigated to find optimal encapsulating materials. The required factors for a polymer gel to be used to encapsulate activated carbon are as follows: non-toxic, insoluble in water, non-degradable by various digestive enzymes, permeable to uremic toxins, and stable over a wide pH range. Bacterial cellulose (BC) gel has good mechanical properties including a high tensile strength and modulus, high water holding capacity, high porosity, high crystallinity, and good biocompatibility [15]. Therefore, BC gel has been studied as a drug carrier for drug delivery systems [16,17] and a scaffold for tissue engineering [18,19]. Previously, we successfully prepared a hollow-type spherical bacterial cellulose (HSBC) gel encapsulating fluorescent particles with diameters of 5.0–5.9 µm larger than the pore size of the BC gelatinous membranes [20]. These encapsulated particles did not leak from the interior cavity of the HSBC gel.

**Figure 1:**
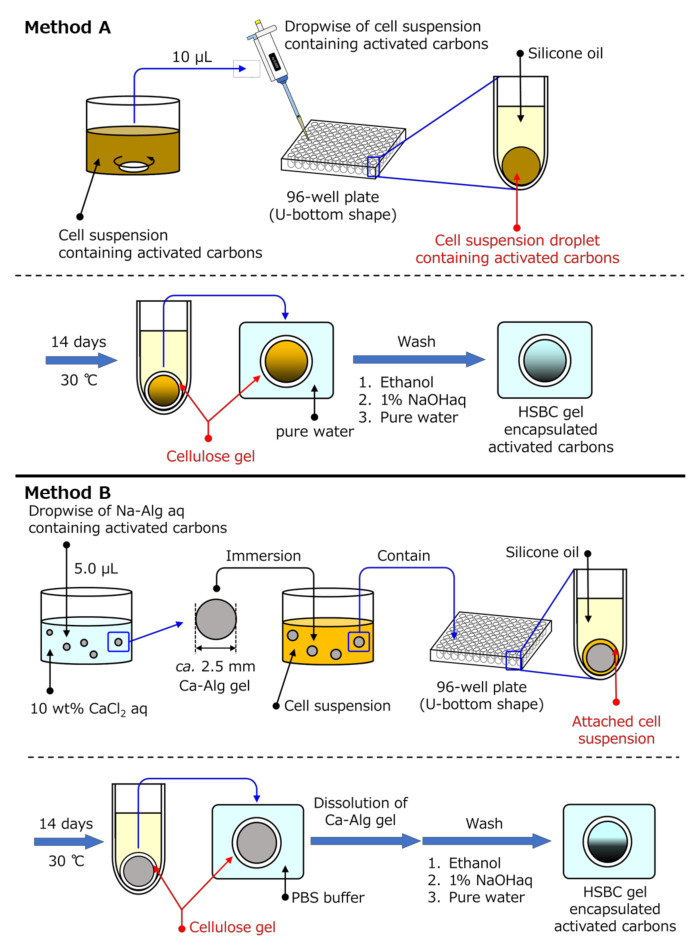
Schematic procedure of Methods A and B for the production of a hollow-type spherical bacterial cellulose (HSBC) gel encapsulating activated carbon.

Here, we attempted to encapsulate activated carbon (with an average diameter of 6 μm) with HSBC gel using a particle-preloaded droplet cultivation method [20]. To achieve this purpose, two encapsulating methods were used. The first method was to produce the BC gelatinous membrane on the interface between silicone oil and Hestrine–Schramm’s medium [21] containing *Komagataeibacter xylinus* (synonym: *Gluconacetobacter xylinus*) and activated carbon (Figure 1, Method A). The second method was encapsulation by forming the BC gelatinous membrane on the surface of the spherical alginate gel (Ca–Alg gel) with a diameter of 2 to 3 mm containing activated carbon (Figure 1, Method B). In the second method, after the production of the membrane, the activated-carbon-encapsulated HSBC gel was obtained by dissolving away the alginate gel. The adsorption ability of the encapsulated activated carbon was evaluated using indole, which is a precursor of the uremic causative agent [22].

## 2. Materials and Methods

### 2.1. Materials

Hestrine–Schramm’s medium (HS medium) [21] was used for the incubation of the bacterial strain. Standard HS medium consisted of a mixture of 3.0 g D-glucose (Kanto Chemical Co. Inc. Chou-ku, Tokyo, Japan), 0.5 g mannitol (Kanto Chemical Co. Inc. Chou-ku, Tokyo, Japan), 0.5 g peptone (HIPOLYPEPTONE^TM^, Nihon Pharmaceutical Co. Ltd. Chou-ku, Tokyo, Japan), 0.5 g Bacto^TM^ yeast extract (BD Biosciences, Franklin Lakes, NJ, USA), and 0.1 g magnesium sulfate heptahydrate (MgSO_4_∙7H_2_O: Kanto Chemical Co. Inc.) in 100 mL Milli-Q water. The density of the HS medium at 30 °C was measured to be 1.02 g/cm^3^ using a Baume hydrometer. Other reagents were purchased from Kanto Chemical Co. Inc. and used as received. Silicone oils (KF-56A: 0.995 g/cm^3^, 15 mm/s^2^, ethanol-soluble oil) were obtained from Shin-Etsu Chemical Co., Ltd. (Chiyoda-ku, Tokyo, Japan). Activated carbon (coconut-shell-activated charcoal, Model No. KD-PWSP, average diameter 6.0 µm, BET surface area 1.324 × 10^3^ m^2^/g) was purchased from AS ONE Corporation(Nishi-ku, Osaka, Japan) and used as received.

### 2.2. Preparation of the Hollow-Type Spherical BC-Gel-Encapsulated Activated Carbon

Figure 1 shows the two preparation methods of the activated carbon-encapsulated HSBC gel. Method A involved producing the BC gelatinous membrane on the interface between silicone oil and a cell suspension containing activated carbon. Method B involved producing the BC gelatinous membrane on the surface of a spherical alginate gel with a diameter of 2 to 3 mm containing activated carbon to be encapsulated in the HSBC gel. The detailed procedures are as follows:

#### 2.2.1. Method A

The HS medium containing 0.5 wt% activated carbon was sterilized by autoclaving and then *Komagateibacter xylinus* (*K. xylinus*, IFO13772) was cultured in the HS medium at 30 °C for 3 days. A 10 µL cell suspension with activated carbon was dropped in each well of a 96-well plate filled with silicone oil. This 96-well plate (Caplugs Evergreen, item: 290-8353-03R) had a U-shaped bottom and was composed of polypropylene (PP). The dropped cell suspension formed a sphere at the bottom of the 96-well plate. While maintaining these states, *K. xylinus* in the cell suspension was cultured for a specified period at 30 °C. The BC gelatinous membrane was produced on the interface of the cell suspension and the silicon oil. After 14 days, the resulting gels were put into excess hot water (>75 °C). The hot water in which the resulting gels were floated was gently agitated for 3 h for sterilization. The remaining silicone oil on the activated-carbon-encapsulated HSBC gel could be removed by this sterilization process. The produced activated-carbon-encapsulated HSBC gel was purified by soaking in a large amount of Milli-Q water for 1 day followed by washing in a 1% (*w*/*v*) aqueous solution of NaOH at room temperature for 1 additional day to remove the bacterial cell debris and alkali-soluble components completely. Then, the activated-carbon-encapsulated HSBC gel was washed in hot water with gentle agitation for at least 3 h to desorb the activated-carbon adsorbates, washed several times with large volumes of Milli-Q water, and stored in Milli-Q water at room temperature.

#### 2.2.2. Method B

Spherical alginate gels (Ca–Alg gel) including activated carbon were prepared by dropping 5 µL of 1 wt% sodium alginate aqueous solution (Na–Alg aq.) suspending 1 wt% or 5 wt% activated carbon into 10 wt% calcium chloride aqueous solution (CaCl_2_ aq.). The obtained Ca–Alg gels were washed by immersion in Milli-Q water.

The HS medium was sterilized by autoclaving, and then *K. xylinus* (IFO13772) was cultured in the HS medium at 30 °C for 3 days. The spherical Ca–Alg gels that included the activated carbon were immersed in the cultured cell suspension and inoculated with *K. xylinus* for one day at 30 °C. Spherical Ca–Alg gels, where the cell suspension remained at the Ca–Alg gel surface, were immersed in each well of a U-shaped bottom 96-well plate filled with silicone oil. While maintaining these states, *K. xylinus* in the cell suspension was cultured for a specified period at 30 °C. With this method, the BC gelatinous membrane was biosynthesized by *K. xylinus* at the interface between the cell suspension attached onto the alginate gel and the silicone oil. After the BC gelatinous membrane was biosynthesized, the alginate gel was dissolved in a phosphate buffer to prepare an HSBC gel with activated carbon. After dissolving away the Ca–Alg, the activated-carbon-encapsulated HSBC gel was purified by the same treatment process as in Method A.

### 2.3. Preparation of HSBC Aerogel Using Supercritical CO_2_

Water-swollen HSBC gel was placed in a large quantity of methanol and washed thoroughly, and the swelling solvent was completely changed from water to methanol. The gel was dried using a supercritical CO_2_ (scCO_2_) technique without disintegrating its microstructure [23]. Drying was conducted under conditions of 40 °C, 20 MPa, and CO_2_ flow rate of 2.0 mL/min for 5 h. The drying apparatus consisted of a CO_2_ delivery pump (SCF-Get, JASCO, Japan), 50 mL pressure vessel, a gas pressure regulator (SCF-Bpg, JASCO, Japan), and a constant-temperature water bath (BK33, Yamato Scientific Co. Ltd., Tokyo, Japan).

### 2.4. Characterization of HSBC Gels

The microstructure of HSBC aerogels was observed using a field-emission scanning electron microscope (FE-SEM: S-4500, Hitachi High-Technologies Corporation, Minato-ku, Tokyo, Japan) with an acceleration voltage of 10 kV. For the pretreatment prior to FE-SEM observation, deposition of Pt–Pd was performed by ion sputtering (E-1010, Hitachi High-Technologies Corporation).

To analyze the composition and the crystal structure of the obtained HSBC aerogels, attenuated total reflection Fourier transform infrared (ATR-FTIR) measurement was carried out. The ATR-FTIR spectra were measured using a FTIR spectrophotometer (Spectrum One, Perkin Elmer, Billerica, MA, USA) equipped with a universal ATR sampling accessory. All measurements were carried out with a nominal spectral resolution of 8 cm^−1^ in transmittance mode and 24 scans.

The weight of encapsulated activated carbon was measured using a microgram balance (Sartorius, MSE3.6P000DM, readability: 1 μg). As a pre-treatment, all samples were dried sufficiently at 50 °C for 1 day. The weight of the activated carbon was determined by the following equation:

Activated carbon (μg) = Activated-carbon-encapsulated HSBC gel (μg) − HSBC gel (μg)(1)

The HSBC gel was prepared on the same day, using the same cell suspension as the preparation of the activated-carbon-encapsulated HSBC gel.

### 2.5. Indole Adsorption Test of Activated Carbon Encapsulated in HSBC Gel

To evaluate the adsorption capacity of the activated- carbon- encapsulated HSBC gel, indole, one of the precursors of the uremic causative agent was used. The adsorption behavior of indole on the activated carbon within the HSBC gel was evaluated by a UV-VIS spectrometer (JASCO Corporation, V-530). Kinetic experiments were performed to determine the equilibrium time of indole adsorption. In the method for quantifying the concentration of indole, absorbance in a wavelength range of 200–340 nm at 37 °C was measured and the concentration of indole was calculated from a preliminarily-formed calibration curve using the indole peak absorbance at 268 nm. The initial concentration of the indole aqueous solution was 5 µg/mL. As described below, the adsorption processes of modeling the uremic toxin, indole, onto the activated carbon were different between native activated carbon and that encapsulated by the HSBC gel. To elucidate this reasons for this, the following calculations were carried out.

#### 2.5.1. Pseudo-First-Order Model

The pseudo-first-order model [24] equation is given as:

(2) $$\frac{dQ_{t}}{dt}=k_{1}\left(Q_{e}-Q_{t}\right)$$

where *Q_t_* is the amount of indole adsorbed at time *t* (mg/g), *Q_e_* is the adsorption capacity at equilibrium (mg/g), *k*_1_ is the pseudo-first-order rate constant (min^−1^), and t is the contact time (min). The integration of Equation (2) with the initial condition, *Q_t_* = 0 at *t* = 0 leads to [24]:

(3) $$Ln\left(Q_{e}-Q_{t}\right)=Ln\;Q_{e}-k_{1}t$$

#### 2.5.2. Pseudo-Second-Order Model

The pseudo-second-order model [25] equation is given as:

(4) $$\frac{dQ_{t}}{dt}=k_{2}{\left(Q_{e}-Q_{t}\right)}^{2}$$

where *k*_2_ is the pseudo-second-order rate constant (g/(mg∙min)). The integration of Equation (4) with the initial condition, *Q_t_* = 0 at *t* = 0 leads to:

(5) $$\frac{1}{\left(Q_{e}-Q_{t}\right)}=\frac{1}{Q_{e}}+k_{2}t$$

Equation (5) can be rearranged to obtain:

(6) $$\frac{t}{Q_{t}}=\frac{1}{k_{2}Q_{e}{}^{2}}+\frac{1}{Q_{e}}t$$

#### 2.5.3. Intra-Particle Diffusion Study

The possibility of intra-particle diffusion was explored by using the intra-particle diffusion model [26,27]:

(7) $$Q_{t}=k_{i}t^{\frac{1}{2}}$$

where *k_i_* is the intra-particle diffusion rate constant (mg∙g^−1^∙min^−1/2^).

## 3. Results and Discussion

### 3.1. Preparation of the HSBC Gel Encapsulating Activated Carbon Using Method A

**Figure 2:**
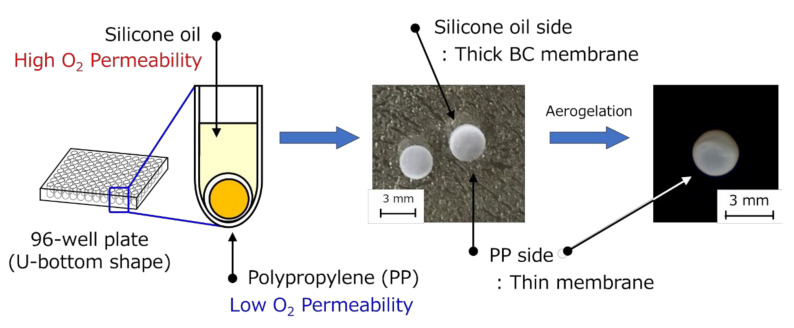
Non-uniformity of the gelatinous membrane thickness of HSBC gel obtained by Method A.

The HSBC gel prepared using Method A had a non-uniform membrane thickness (Figure 2). In the thick membrane region, *K. xylinus* was cultured at the interface between the cell suspension and the silicone oil. Where the cell suspension was in contact with the bottom of a 96-well plate composed of polypropylene (PP), the BC gelatinous membrane was thin. The difference in film thickness is due to the difference in oxygen permeability between silicone oil and PP. *K. xylinus* are aerobic bacteria and thus require oxygen to produce cellulose [28]. Therefore, cellulose was likely to be biosynthesized on the side of the silicone oil side that had a higher oxygen permeability than PP, resulting in the BC gelatinous membrane being thicker.

**Figure 3:**
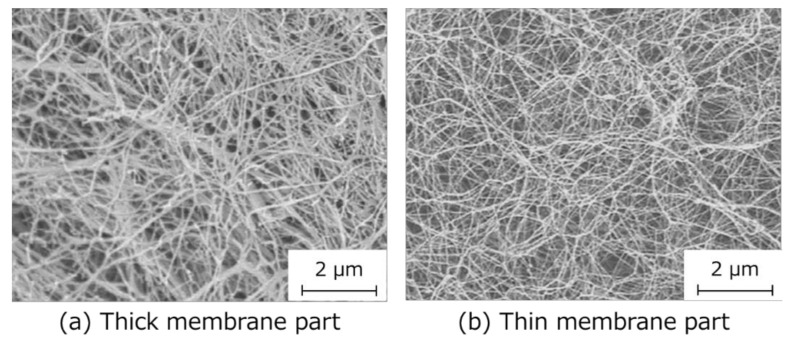
Scanning electron microscopy (SEM) images of HSBC aerogel. (**a**) The surface of the thick membrane and (**b**) the surface of the thin membrane.

SEM observations of HSBC aerogels showed a network structure of cellulose nanofibers in both thick and thin parts of the BC membrane (Figure 3). In the thicker part of the BC gelatinous membrane, the diameter of the cellulose nanofibers was about 30 nm. On the other hand, the cellulose nanofiber of the thin part of the BC gelatinous membrane may have had a smaller diameter than that. Cellulose nanofibers were readily produced at the interface between the silicone oil and the cell suspension, and so cellulose nanofibers may have been more likely to aggregate with each other. The common feature of both was that the cellulose nanofibers formed a fine network structure with a pore size of less than 1 µm.

**Figure 4:**
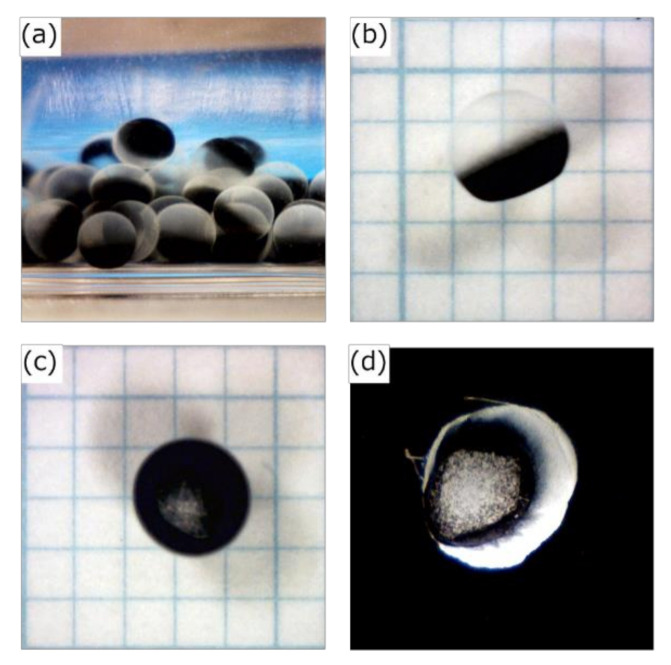
Photographs of activated-carbon-encapsulated HSBC gel: (**a,b**) side view, (**c**) bottom of the activated-carbon-encapsulated HSBC gel, and (**d**) bottom of the activated-carbon-encapsulated HSBC aerogel after supercritical drying.

Activated-carbon-encapsulated HSBC gel is shown in Figure 4. Activated carbon was non-uniformly present on one side of the HSBC gel interior (Figure 4a,b). This was due to the fact that the activated carbon precipitated over time during the cultivation period. Even though the activated-carbon-encapsulated HSBC gel was subjected to external forces such as agitation, the encapsulated activated carbon remained immobile inside the HSBC gel. This fact suggests that encapsulated activated carbon was adsorbed or entangled in the cellulose nanofiber network. Furthermore, the resulting HSBC gel had areas without activated carbon (Figure 4c). After scCO_2_ drying, the membrane was observed in the area without activated carbon (Figure 4d).

**Figure 5:**
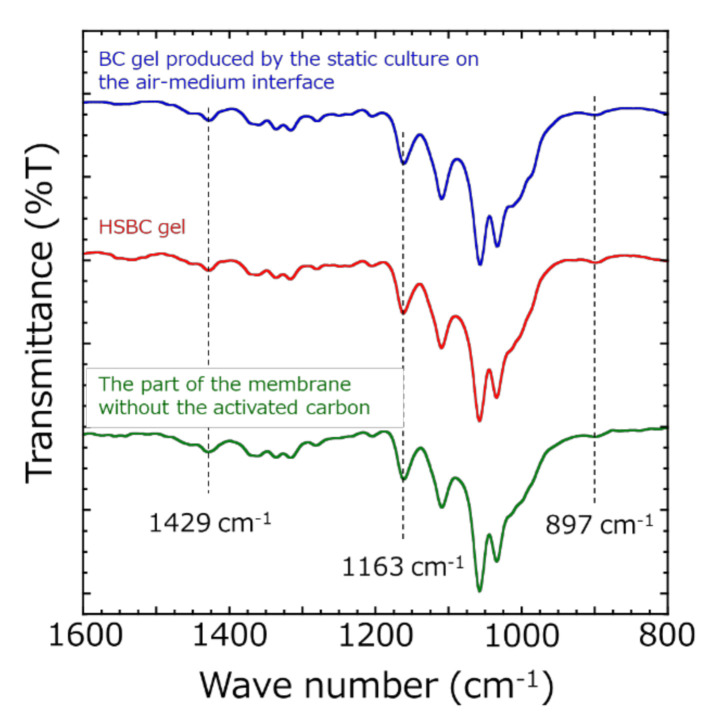
Attenuated total reflection Fourier transform infrared (ATR-FTIR) spectra of the parts of the membrane without activated carbon after scCO_2_ drying: HSBC aerogel and conventional bacterial cellulose (BC) aerogel.

ATR-FTIR measurements of the part of membranes without activated carbon after scCO_2_ drying were performed (Figure 5). As a comparative sample, the BC gel obtained from the test tube and the HSBC gel were presented. These samples were dried by scCO_2_ with the same procedure. All samples showed nearly identical spectra and the absorptions at 1429 cm^−1^ (CH_2_ bending), 1163 cm^−1^ (C-O-C stretching), and 897 cm^−1^ (β-glucosidic linkage) that are specific to cellulose type I crystals [29] were confirmed. *K. xylinus* biosynthesized the BC gelatinous membrane at the air–medium interface in static culture method. The ATR-FTIR spectrum of HSBC gel were consistent with the results of the static culture method, revealing that *K. xylinus* biosynthesized the BC gelatinous membrane at the silicone oil–medium interface. Furthermore, the ATR spectrum of the part of the membrane without activated carbon in Figure 4d agreed with the HSBC gel, indicating that these parts was formed by the BC gelatinous membrane.

**Figure 6:**
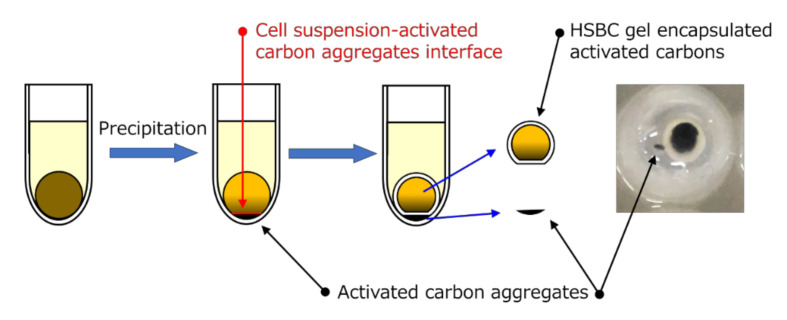
Schematic representation of the formation process of the BC gelatinous membrane without activated carbon.

The formation of a BC gelatinous membrane without activated carbon was considered to be due to the following factors. Figure 6 shows the formation process of the BC gelatinous membrane without activated carbon. Approximately 3 days after the start of culturing, activated carbon in the cell suspension droplets precipitated and aggregated. The BC gelatinous membrane was produced at the interface between the cell suspension and activated-carbon aggregates. Consequently, observing the 96-well plate immediately after cultivation, non-encapsulated activated carbon could be confirmed. The amount of encapsulated activated carbon within the HSBC gel by Method A (32.6 ± 7.1 μg) was lower than the calculated amounts (50.0 μg). The calculated amount of encapsulated activated carbon was obtained by calculating the activated carbon contained in 10 µL of a cell suspension containing 0.5 wt% of activated carbon (see Section 3.2, Table 1).

### 3.2. Preparation of the HSBC Gel Encapsulating Activated Carbon Using Method B

Method A could not encapsulate a large amount of activated carbon. The reason for this is that activated carbon precipitated and aggregated during the culture period. In Method B, the Ca–Alg gel was used to prevent the precipitation of activated carbon during the culture period. The details of the HSBC gel obtained by Method B have been reported previously [20], and the gelatinous membrane was formed by a network structure of cellulose nanofibers with a diameter of about 30 nm and a pore size of less than 1 µm. The structure of the BC gelatinous membrane of HSBC gel obtained by Method B was similar to that obtained by Method A. The structure of the HSBC gelatinous membrane obtained by Method B was expected to encapsulate 6 µm of activated carbon.

**Figure 7:**
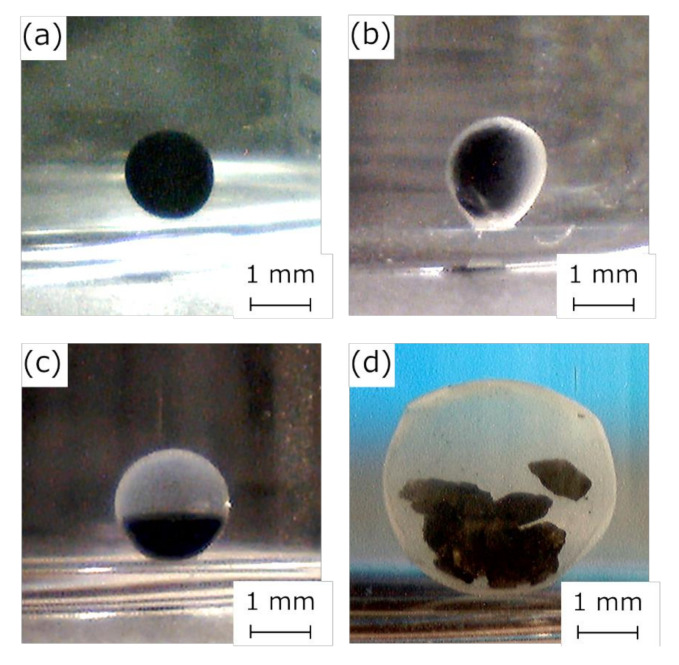
Photographs of (**a**) Ca–Alg gel containing 5 wt% activated carbon, (**b**) activated-carbon-encapsulated HSBC gel before dissolving away Ca–Alg gel, (**c**) activated-carbon-encapsulated HSBC gel, and (**d**) a large diameter activated-carbon-encapsulated HSBC gel.

Figure 7 shows photographs of the activated carbon-encapsulated HSBC gel prepared by Method B. The activated-carbon-encapsulated HSBC gel obtained by Method B was formed with a uniform BC gelatinous membrane (Figure 7c). The encapsulated activated carbon was non-uniformly present on one side of the HSBC gel, with the same results as Method A. In the Ca–Alg gel containing activated carbon (Figure 7a), the activated carbon was uniformly dispersed. Before the Ca–Alg gel was dissolved away (Figure 7b), a thin white membrane was formed around the black Ca–Alg gel, and the inside was uniformly black, meaning that the activated carbon was uniformly distributed. Therefore, by dissolving away the Ca–Alg gel with PBS, it was considered that activated carbon precipitated in the HSBC gel and was adsorbed on the inner surface of the HSBC gel. In Method B, activated carbon with a large particle size (coconut-shell-activated charcoal, Model No. KD-GW-A, 20 × 50 mesh) was also successfully encapsulated within the HSBC gel (Figure 7d). In the encapsulation of large particles by Method A, accurate volume control was difficult because the pipette was easily clogged when the cell suspension containing activated carbon was aspirated and dropped. No leakage of these encapsulated activated carbon has been confirmed even after storage in Milli-Q water for more than a month.

**Figure 8:**
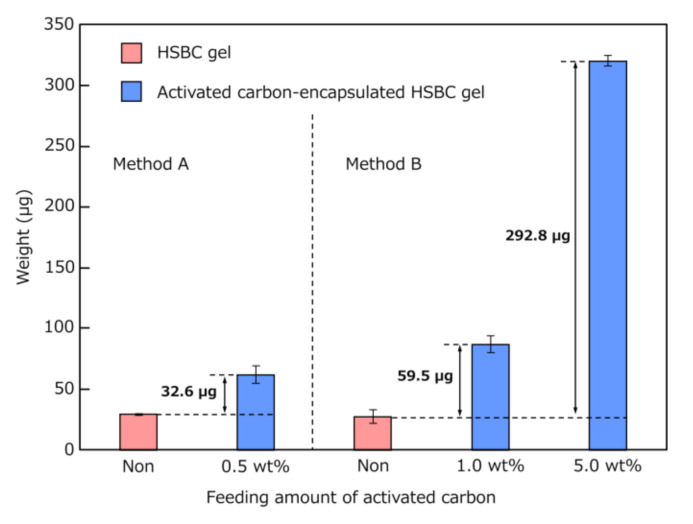
Dry weight of HSBC gel and activated carbon encapsulated in HSBC gel (*n* > 6).

Figure 8 and Table 1 show the results regarding the amount of encapsulated activated carbon. Comparing the calculated values with the measured values, the measured values in Method A were lower than the calculated values. As explained in Figure 6, the reason for this result was that parts of the activated carbon precipitated during the cultivation period were not encapsulated. In Method B, the amount of encapsulated activated carbon was higher than the calculated value, suggesting that all the feeding-activated carbon was encapsulated. Method B successfully encapsulated more activated carbon than Method A. In the comparison of the activated-carbon-encapsulated HSBC gel by Method B, the encapsulating amount of activated carbon using Na-Alg aqueous solution containing 5 wt% activated carbon was 4.92 (≈ five times larger than that of 1 wt%). This result indicated that the amount of encapsulation depended on the feeding amount of activated carbon. In Method A, using the same amount of activated carbon as the 5 wt% of Method B, the growth of *K. xylinus* was inhibited, meaning that the activated-carbon-encapsulated HSBC gel could not be obtained. Therefore, Method B is more suitable for the encapsulation of large particle sizes or a large amount of activated carbon within the HSBC gel.

### 3.3. Indole Adsorption Test

An adsorption test of indole, which is a precursor of the uremic toxin, on the activated carbon encapsulated in HSBC gel was performed at 37 °C. The initial concentration of indole was 5.0 µg/mL [30]. In the adsorption test, three pieces of the activated- carbon-encapsulated HSBC gel (total activated carbon: 32.6 × 3 = 97.8 µg) obtained by Method A were added to a 3 mL indole aqueous solution. On the other hand, an adsorption test was carried out with one activated-carbon-encapsulated HSBC gel (activated carbon: 292.8 µg) obtained by Method B.

**Figure 9:**
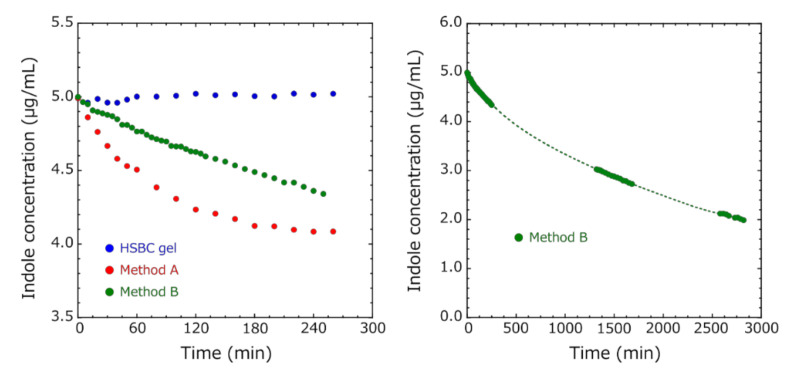
The variation of indole concentration over time. Adsorption conditions: indol concentration = 5.0 µg/mL, temperature = 37 ℃, added HSBC gel = three pieces, added activated-carbon-encapsulated HSBC gel (Method A) = three pieces, added activated-carbon-encapsulated HSBC gel (Method B) = one piece).

Figure 9 shows the variation of indole concentration over time. In the HSBC gel, since there was almost no change from the initial concentration, it was clarified that indole adsorption did not occur. In Method A, the adsorption equilibrium was reached in a short time with a small decrease in indole concentration. The amount of adsorbed indole that was calculated from the indole concentration at 260 min was 0.91 μg per the activated-carbon-encapsulated HSBC gel. The adsorption capacity at equilibrium Q_e_ (mg/g) of indole was 27.8 mg/g. In Method B, adsorption equilibrium was not reached even after 48 h. However, the amount of encapsulated activated carbon increased, so the amount of indole adsorbed increased. At 2820 min, 9.03 μg of indole per activated carbon encapsulated in HSBC gel was adsorbed. These facts suggested that the amount of adsorbed indole depends on the amount of encapsulated activated carbon within the HSBC gel. Moreover, Methods A and B used activated carbon with the same particle size, but the adsorption rate of Method A was faster. Since adsorption occurred only inside the HSBC gel, these results suggested that Method A with three pieces of the activated-carbon-encapsulated HSBC gel was more likely to contact with the encapsulated activated carbon and indole molecules.

### 3.4. Adsorption Kinetic Study

**Figure 10:**
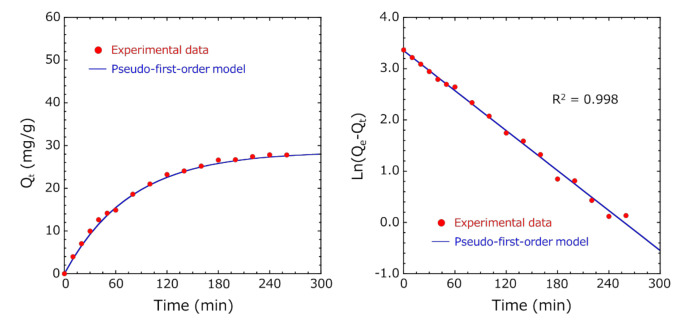
Fitting of the pseudo-first-order model for indole adsorption on encapsulated activated carbon within HSBC gel (Method A). (**Left**) plot of adsorbed capacity versus time and (**right**) pseudo-first-order adsorption kinetics.

To explore the adsorption mechanism and the adsorption capacity, the kinetic data were fitted with pseudo-first-order, pseudo-second-order, and intraparticle diffusion models [24,25,26,27]. Q_e_ (mg/g) of indole was calculated by fitting the adsorption results using the pseudo-first-order reaction model [24,25]. Figure 10 shows the fitting results of Method A. In pseudo-first-order absorption kinetics, the Ln(*Q_e_* − *Q_t_*) vs. time plot was fitted so that the R^2^ value was the highest, and Q_e_ was calculated. The pseudo-first-order model showed an excellent correlation of R^2^ = 0.998, and Q_e_ was 28.6 mg/g, which was almost identical to the value (27.8 mg/g) obtained from the indole concentration at 260 min. This result suggests that the diffusion of indole is a rate-limiting step for adsorption to encapsulated activated carbon.

**Figure 11:**
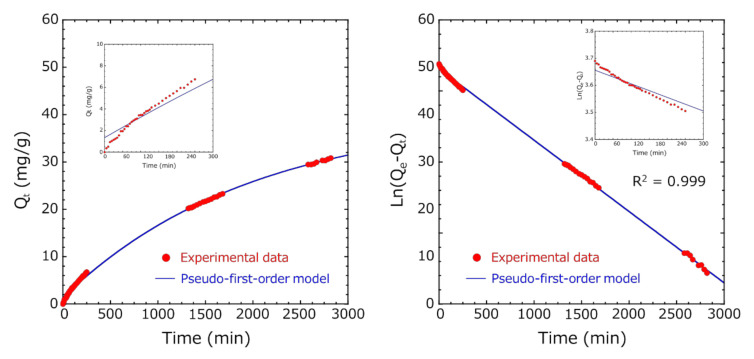
Fitting of the pseudo-first-order model for indole adsorption on encapsulated activated carbon within HSBC gel (Method B). (**Left**) plot of adsorbed capacity versus time and (**right**) pseudo-first-order adsorption kinetics.

**Figure 12:**
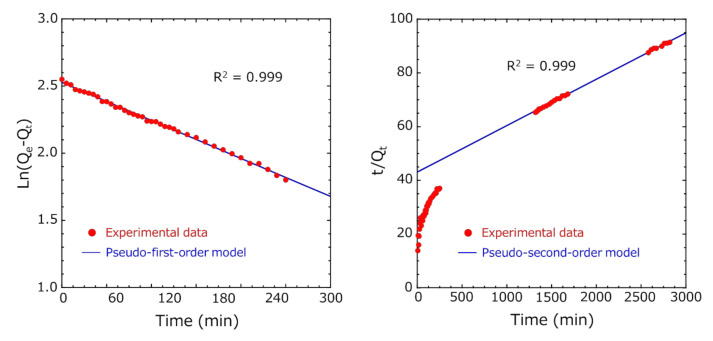
Fitting of adsorption kinetics of indole adsorption on encapsulated activated carbon within HSBC gel (Method B). (**Left**) pseudo-first-order model at the initial stage and (**right**) pseudo-second-order model at the later stage.

Similarly, in Method B, in the pseudo-first-order absorption kinetics, the Ln(*Q_e_* − *Q_t_*) vs. time plot was fitted so that the R^2^ value was the highest in the entire measurement range until 2820 min, but the initial fitting accuracy of adsorption was low (Figure 11). Ho et al. reported that the pseudo-first-order model fits well in the initial stages of adsorption, as the adsorption amount increases sharply [25]. Many researchers have analyzed various adsorbent and adsorbate combinations using the pseudo-second-order model [31,32]. Therefore, fitting was performed using the pseudo-first-order model in the initial stage of adsorption and the pseudo-second-order model in the later stage of adsorption (Figure 12). By dividing the total into two regions, we succeeded in fitting (R^2^ = 0.999) both the initial and later stages of adsorption very well. The adsorption capacity at equilibrium Q_e1_ of the pseudo-first-order model was 12.8 mg/g, and the adsorption capacity at equilibrium Q_e2_ of the pseudo-second-order model was 57.9 mg/g.

However, the pseudo-second-order model assumes that the rate-limiting step may be chemical adsorption, involving valency forces through sharing or exchange of electrons between adsorbent and adsorbate [25]. Since both Methods A and B followed the pseudo-first-order model at the initial stage of adsorption, the adsorption process of encapsulated activated carbon and indole was physical adsorption. Thus, it is unlikely that the adsorption mechanism of Method B changed to chemical adsorption during the adsorption process. Recently, many researchers have reported that a diffusion-controlled process is better described by the pseudo-second-order model than the pseudo-first-order model, although this model cannot represent the steep rise of the uptake at short times [33]. Furthermore, results well described by the pseudo-secondary model show that diffusion (outer layer and/or intraparticle) contributes significantly [34].

**Figure 13:**
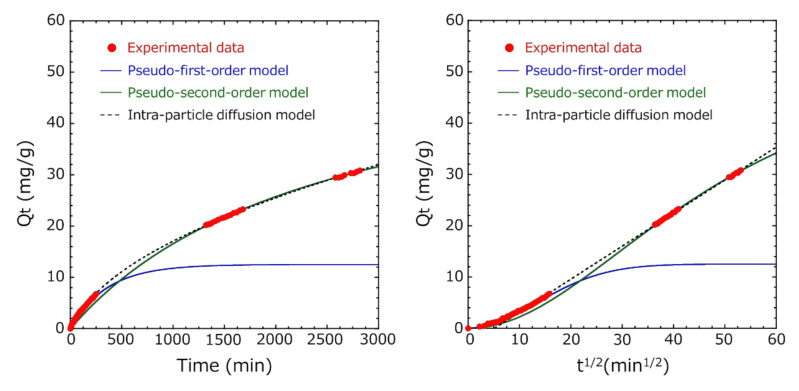
(**Left**) The effect of the contact time on the adsorption of indole by encapsulated activated carbon within the HSBC gel and (**right**) intra-particle diffusion model curve of indole adsorption (R^2^ = 0.999 (*t*^1/2^ > 10 min^1/2^)).

Therefore, the adsorption mechanism was investigated using an intra-particle diffusion model [26,27]. As seen in Figure 13 (right), a linear relation existed between *Q_t_* and *t*^1/2^ over a wide range after *t*^1/2^ = 10 min^1/2^, suggesting the presence of an external mass transfer resistance during indole adsorption. From the above, after the exterior surface of the encapsulated activated carbon within the HSBC gel obtained by Method B reached adsorption saturation following the pseudo-first-order model, the indole molecules were impregnated into the intra-particles of the activated carbon, where they were adsorbed.

These results suggested that the diffusion of the indole molecules on the surface of the encapsulated activated carbon within HSBC gel was dominant at the initial stage of adsorption, and as the adsorption time became longer, the adsorption by the intra-particle diffusion process of the encapsulated activated carbon became dominant.

### 3.5. Comparison of the Adsorption Capacity at Equilibrium of the Activated-Carbon-Encapsulated HSBC Gel Obtained by Methods A and B

In the activated-carbon-encapsulated HSBC gel obtained by Method A, the adsorption capacity at equilibrium Q_e_ was 28.6 mg/g. In Method B, the adsorption capacity at equilibrium Q_e_ was 57.9 mg/g and was larger than that of Method A. As discussed in Section 3.4, the validity of the pseudo-second-order model fitting needs to be verified, and the comparison of the results of the pseudo-primary model and the pseudo-secondary model may be inappropriate. However, it is clear that Method B had a larger adsorption capacity at equilibrium Q_e_ than Method A, as shown in Figure 11, Figure 12 and Figure 13. This difference in the adsorption capacity at equilibrium was considered to be attributed to the difference in the encapsulation process. In Method A, activated carbon was added directly to the cell suspension for encapsulation within the HSBC gel. Thus, the activated carbon adsorbed organic substances and various ions in the cell suspension during the 14-day culture period. This was considered to reach adsorption equilibrium. In the desorption process (Section 2.2.1), adsorbates desorbed from the outer surface only, which was the easy desorption area of activated carbon, and the adsorption active site existed only on the outer surface of the activated carbon. Thus, this suggests that the experimental data are in good agreement with the pseudo-first-order model that depends on the diffusion of indole onto the outer surface of activated carbon.

In Method B, solutes in the cell suspension that permeated the Ca–Alg gel might have been adsorbed on the activated carbon. The amount of cell suspension was less than in Method A; the permeated solutes were more adsorbed on the outer surface than the inner surface of the activated carbon. Since the active adsorption site of the inner surface of the activated carbon remained, the adsorption of activated-carbon-encapsulated HSBC gel obtained by Method B was dominated by the intra-particle diffusion process.

### 3.6. Influence of BC Gelatinous Membrane on Indole Adsorption

The indole molecules that permeated the BC gelatinous membrane could adsorb onto the encapsulated activated carbon within the HSBC gel. If there was an interaction between the BC gelatinous membrane and the indole molecules, there should have been effects such as a time lag before the start of adsorption. The following two results were considered to suggest that the BC gelatinous membrane did not affect indole adsorption:

1. The HSBC gel without activated carbon did not adsorb indole molecules.
2. In both Methods A and B, the diffusion of indole molecules on the outer surface of activated carbon was dominant at the initial stage of adsorption because these experimental data fitted well with the pseudo-first-order model.

The molecular radius, *r*, of indole that was assumed to be a sphere was calculated using the following equation:

(8) $$\frac{4}{3}\pi r^{3}=\frac{M_{w}}{d\cdot N_{A}}$$

where *M_w_* is the molecular weight of indole (117.15 g/mol), d is the density of indole (1.22 g/cm^3^), and *N_A_* is Avogadro’s constant. The molecular radius of indole is 0.34 nm. The molecule size of indole is much smaller than the distance between the cellulose nanofibers forming the gelatinous membrane of HSBC gel (see Figure 3). This result indicates that the BC gelatinous membrane does not interfere with the permeation of indole molecules. If the adsorbates with a small molecular size do not adsorb onto cellulose nanofibers, this suggests that the BC gelatinous membrane does not affect the adsorption process on the encapsulated activated carbon within HSBC gel.

## 4. Conclusions

We have succeeded in preparing the activated-carbon-encapsulated HSBC gel by two different methods. The encapsulated activated carbon could neither pass through the BC gelatinous membrane of the HSBC gel nor leak from the interior cavity of the HSBC gel. In Method A, where activated carbon was added directly to the cell suspension, a small amount of activated carbon was encapsulated. On the other hand, Method B, which used Ca–Alg gel containing activated carbon, succeeded in controlling the amount of encapsulated activated carbon by the amount of feeding-activated carbon. In the adsorption test, the activated carbon-encapsulated HSBC gel prepared by Methods A and B adsorbed indole. No adsorption of indole was observed on the cellulose nanofibers forming the HSBC gel. In addition, the BC gelatinous membrane did not affect the adsorption process on the encapsulated activated carbon within the HSBC gel. The adsorption followed the pseudo-first-order model and intra-particle diffusion model. These results suggested that the diffusion of the indole molecules on the surface of the encapsulated activated carbon within the HSBC gel was dominant at the initial stage of adsorption, and that as the adsorption time became longer, the adsorption by the intra-particle diffusion process of the encapsulated activated carbon became dominant.

HSBC gel with excellent biocompatibility, mechanical properties, high wettability, acid and base resistance, and selective permeability by size exclusion could be suitable for the encapsulation of oral formulations of activated carbon. Although tests regarding the living environment, such as tests for pH, ion concentration, viscosity, and the presence of digestive enzymes are not sufficient, HSBC-gel-encapsulating-activated carbon may provide an improved ease of swallowing, and a BC gelatinous membrane may prevent the leakage of encapsulated activated carbon in the oral and intestinal environment. Furthermore, encapsulation with HSBC gel by the particle-preloaded droplet cultivation method using Ca–Alg gel can encapsulate particles regardless of whether they are organic or inorganic substances [20]. In addition to activated carbon, various oral adsorbents are used clinically. Cation or anion exchange resins and inorganic microporous materials are used as oral adsorbents for uremia, hyperkalemia, and hypercholesterolemia. Similarly to activated carbon, the adsorption capacity of these oral adsorbates increases as the particle size decreases, but the smaller particle size facilitates adsorption to the gastrointestinal wall, and these adsorbed particles induce serious side effects. Payne et al. have reported in calf experiments that small particles (<5 μm) are absorbed through the mucosa and deposited in reticuloendothelial tissues [35]. All of these problems result from the contact of oral adsorbates with the gastrointestinal wall. The encapsulation of these oral adsorbents with HSBC gel may achieve the adsorption of the target substance in the gastrointestinal tract without adsorbing onto the gastrointestinal wall.

## Figures and Tables

**Table 1:** Comparison of calculated and measured values of encapsulated activated carbon.

Activated Carbon	Dropwise Volume	Encapsulated Activated Carbon	
(wt%)	(µL)	Calculated (µg)	Measured (µg)
0.5 (Method A)	10.0 *	50.0	32.6
1.0 (Method B)	5.0 **	50.0	59.5
5.0 (Method B)	5.0 **	250.0	292.8

* Method A: cell suspension containing activated carbons. ** Method B: Na-Alg aqueous solution containing activated carbons.
